# New Insights into Aspirin’s Anticancer Activity: The Predominant Role of Its Iron-Chelating Antioxidant Metabolites

**DOI:** 10.3390/antiox14010029

**Published:** 2024-12-29

**Authors:** George J. Kontoghiorghes

**Affiliations:** Postgraduate Research Institute of Science, Technology, Environment and Medicine, Limassol 3021, Cyprus; kontoghiorghes.g.j@pri.ac.cy; Tel.: +357-26-272-076

**Keywords:** aspirin, antioxidants, anticancer effects, iron-chelating metabolites, salicylic acid, salicyluric acid, genticic acid, 2,3-dihydroxybenzoic acid

## Abstract

Epidemiological studies have suggested that following long-term, low-dose daily aspirin (LTLDA) administration for more than 5 years at 75–100 mg/day, 20–30% of patients (50–80 years old) had a lower risk of developing colorectal cancer (CRC) and about the same proportion in developing iron deficiency anemia (IDA). In cases of IDA, an increase in iron excretion is suspected, which is caused by aspirin chelating metabolites (ACMs): salicylic acid, salicyluric acid, 2,5-dihydroxybenzoic acid, and 2,3-dihydroxybenzoic acid. The ACMs constitute 70% of the administered aspirin dose and have much longer half-lives than aspirin in blood and tissues. The mechanisms of cancer risk reduction in LTLDA users is likely due to the ACM’s targeting of iron involved in free radical damage, iron-containing toxins, iron proteins, and associated metabolic pathways such as ferroptosis. The ACMs from non-absorbed aspirin (about 30%) may also mitigate the toxicity of heme and nitroso-heme and other iron toxins from food, which are responsible for the cause of colorectal cancer. The mode of action of aspirin as a chelating antioxidant pro-drug of the ACMs, with continuous presence in LTLDA users, increases the prospect for prophylaxis in cancer and other diseases. It is suggested that the anticancer effects of aspirin depend primarily on the iron-chelating antioxidant activity of the ACMs. The role of aspirin in cancer and other diseases is incomplete without considering its rapid biotransformation and the longer half-life of the ACMs.

## 1. Introduction

Cancer is a major health problem affecting all countries worldwide, causing many economic, social, and other adverse implications [1]. Many public campaigns for the prevention of cancer and new diagnostic and therapeutic advances have been taking place in the last few years. However, despite all these efforts, it is estimated that there are about 20 million new cancer cases and 10 million related deaths each year, which overall classifies this disease in the top group of incurable and fatal diseases affecting humans [2,3,4]. The design of new therapeutic and preventative strategies against cancer could save millions of lives worldwide. In this context, epidemiological and other studies including the assessment of many generic and experimental drugs receive emergency approval for rapid clinical testing in cancer, because of the low rate of success with many of the current available anticancer therapies [5].

Aspirin, or more precisely its active ingredient acetylsalicylic acid, is one of the oldest generic drugs, which has been used clinically since about 1900 [6]. It is also one of the most commonly used drugs, which is inexpensive and widely available worldwide in different formulations and can be bought over pharmacy counters or prescribed by physicians [6,7]. It has many uses including analgesic, anti-inflammatory, antipyretic, and antithrombotic activity [6,7,8]. The posology of acetylsalicylic acid (thereafter named as aspirin) varies in each condition. It can range from about 75–200 mg/day, which is used for prophylaxis by millions of the aged (>50 years) population for anti-platelet activity and cardiovascular complications, to about 300–600 mg every 4–6 h for analgesic function [9,10,11,12,13]. The principal mechanism of action of aspirin is considered to be the irreversible inhibition of the iron-containing enzymes cyclo-oxygenases (COX-1 and COX-2), which are involved in prostaglandin biosynthesis and platelet aggregation [8,14,15,16].

The pharmacological and metabolic properties of aspirin have been previously described [17,18]. Briefly, it has been estimated that orally administered aspirin, for example at the low dose of 75–100 mg/day, is highly bioavailable, with about 60–70% of the drug being absorbed from the gastrointestinal tract (GIT). Most of the aspirin is absorbed within minutes, mainly from the stomach and upper small intestine, and is also rapidly cleared from the blood circulation with a half-life of about 15–20 min [19,20]. The aspirin concentration achieved in plasma varies according to the administered dose, with, for example, the dose of about 75 mg reaching 7.3 μM, the dose of 325–600 mg reaching 25–80 μM, and the dose of 1.2 g reaching 144 μM [17]. Aspirin is also almost completely metabolized (hydrolyzed/deacetylated) in the GIT, blood, and liver to salicylic acid, which is the predominant initial major metabolite in plasma [21,22,23]. The concentration of salicylic acid in plasma has been estimated to be about 15 μM for the 75 mg dose, 500 μM for the 325–600 mg doses, and 1.5–2.5 mM for the 1.2 g dose [17]. Salicylic acid is then further metabolized to other metabolites, all of which are cleared through the kidneys and excreted in the urine [18,19,20,24,25].

In contrast to aspirin, salicylic acid has a much longer half-life of about 2–6 h and is also mostly metabolized in the liver, with only about 10% of unchanged salicylic acid being excreted in the urine [18,19,20,21,22,23,26,27,28]. There are in total five metabolites of salicylic acid produced in different amounts, two glucuronide conjugates (salicylphenol glucuronide and salicylacyl glucuronide) totaling to about 15%, two dihydroxybenzoic acids (2,3-dihydroxybenzoic acid and 2,5-dihydroxybenzoic acid) totaling to about 2–3%, and salicyluric acid, which is by comparison the metabolite excreted in the urine in the highest proportion, amounting to about 70% [18,19,20,22,23,26,27,28,29,30,31,32].

There are many factors in addition to the aspirin dose that could affect the level of aspirin, salicylic acid, and each of its other metabolites in the blood plasma and urinary excretion of treated patients, including absorption, distribution, metabolism, excretion, toxicity (ADMET) patient characteristics, age, gender, organ function, interactions with food substances, and interactions with other drugs [18,21,22,23,27,31,33,34,35,36].

**Figure 1:**
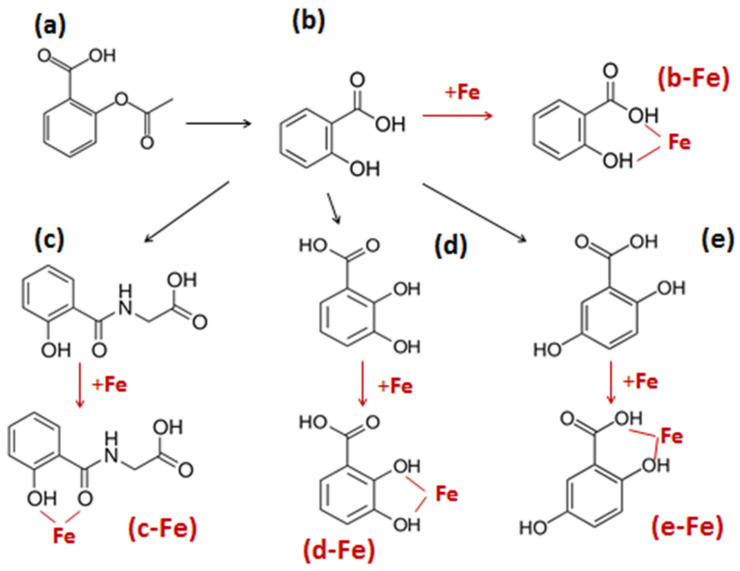
The metabolic transformation of aspirin to iron-chelating metabolites. Aspirin (acetyl salycilic acid) (a) is initially metabolized to salicylic acid (b) and then to several other metabolites including salicyluric acid (c), 2,3-dihydroxybenzoic acid (d), and 2,5-dihydroxybenzoic acid (genticic acid) (e), all of which have strong iron-binding properties. The iron-binding sites and iron complex formation are shown in red color for each of aspirin’s chelating metabolites (b-Fe, c-Fe, d-Fe, and e-Fe).

One of the major findings regarding the pharmacological activity of aspirin is the occurrence of secondary clinical effects, which in addition to the main clinical effects described above have been observed in many patients and in many follow-up epidemiological studies using long-term, low-dose daily aspirin (LTLDA) for prophylaxis. In particular, it has been observed that following the use of LTLDA for usually more than 5 years at 75–100 mg/day, there is an increasing risk of developing iron deficiency anemia (IDA) in the absence of major gastric bleeding [37,38,39]. This secondary clinical effect (IDA) was not observed in similar groups of either non-aspirin users or users of other anti-inflammatory drugs [37,38,39]. It has been recently proposed that a possible cause for IDA in LTLDA users in the absence of major gastric bleeding is the increase in iron excretion associated with the aspirin chelating metabolites (ACMs), which are salicylic acid, salicyluric acid, 2,5-dihydroxybenzoic acid, and 2,3-dihydroxybenzoic acid, all of which amount to about 60–70% of the administered aspirin dose (Figure 1) [18]. Similarly, the affinity for iron binding and iron complex formation, as well as the associated properties and effects of each of the ACMs, has also been reviewed in the same proposal with emphasis on their iron mobilization effects and body iron loss mechanisms in relation to the cause of IDA (Figure 1) [18].

Another major secondary clinical finding following LTLDA use, which received major publicity and attention from patient groups, investigators, physicians, and regulatory authorities, is the reduction in the incidence of cancer [40,41,42]. The link of LTLDA with the decreased incidence of cancer is currently a major worldwide health issue considering that millions of elderly people, including, for example, 50% of older individuals in the USA, are using this prophylactic treatment against many diseases including cardiovascular and cerebrovascular disease and cancer [18,37,38,39,40,41,42,43]. In particular, the identification and acknowledgement of the reduction in the risk of the development of cancer led the USA health authorities to recommend the use of LTLDA for the chemoprevention of colorectal cancer in patients 50–59 years of age [44,45,46,47]. The exact mechanism of the anticancer activity and body iron reduction in LTLDA users is still unclear, and further investigations are in progress [40,41,42,48,49,50,51,52].

The aim of this review is to examine the mechanistic insights and impact of LTLDA use in relation to cancer prevention and the possible anticancer potential of aspirin and the ACMs, including iron-chelating capacity and antioxidant potential, with emphasis on the targeting and inhibition of specific iron metabolic pathways, iron proteins, and other iron processes involved in colorectal and other cancers. Furthermore, it examines other factors that may influence the anticancer activity of aspirin and the ACMs, as well as suggests possible therapeutic and other interventional strategies, such as combination therapies, optimal aspirin posology, and other mechanisms or procedures that may enhance anticancer therapeutic outcomes and reduce the toxicity implications in different categories of LTLDA users.

## 2. General Pharmacological Aspects of the Anticancer Activity of the Aspirin Iron-Binding Metabolites

There are many thousands of preclinical and clinical publications in the scientific literature related to aspirin and cancer, including several thousands of reports on aspirin and anticancer activity. These reports describe many in vitro, in vivo, and clinical studies, including pharmacological and epidemiological findings in different categories of patients using LTLDA [8,18,33,44,47].

Despite that the original suggestions of the possible link between aspirin use and anticancer activity were introduced about 50 years ago, there are still many, but not widely accepted theories, unanswered questions, and different opinions on the usefulness of aspirin in cancer [53,54,55]. In particular, there are especially conflicting views on epidemiological findings in many fields related to the extent of the level of benefit of aspirin use for preventing and reducing the growth of different types of cancer [18,33,44].

The limitations affecting the link between aspirin use and anticancer activity are extended in other related fields, including the lack of specified and widely accepted aspirin anticancer mechanisms of action and associated pharmacological activity parameters. These parameters include, for example, active component(s) and target(s) of aspirin and metabolites, effective doses, durations of the administration of aspirin for achieving anticancer effects, toxicity risk/benefit assessments of LTLDA use in cancer patients, synergistic and antagonistic effects from other drugs and dietary molecules, and the level of impact on different types of cancer, metastasis, drug resistance, etc. [21,33,34,47,49,51].

One of the most important aspects of the pharmacological activity of aspirin, including the possible anticancer implications, is the impact and effect of its metabolites, especially considering that aspirin absorbed from the GIT is rapidly and completely metabolized [18,33,56,57]. In particular, the effects and implications of the different ACMs, which constitute the major quantitative component of the aspirin administered oral doses, may play a crucial role not only in iron depletion in IDA but also in anticancer activity [18,51,58]. This mechanistic proposal was initially suggested because the ACMs appear to possess high iron-binding affinity and because of their involvement in a wide range of interactions with iron-containing proteins and iron metabolic pathways, as well as antioxidant activity and related effects [18].

The link between iron and cancer is another major area of long-term investigations and assessment for targeting purposes and therapeutic interventions [59,60]. Iron is an essential micronutrient not only for normal cells but also for the growth and proliferation of cancer cells [59,60,61,62]. The requirements for iron supply for each cancer cell type is different, similar to the requirements observed for each type of normal cells [59,60,61,62]. Furthermore, iron is the major biological catalyst for free radical formation and cascades, which may lead to molecular, sub-cellular, cellular, and tissue damage, as well as DNA damage and cancer formation [63,64,65,66,67,68]. Most importantly, the essential role of iron in ferroptotic programmed cell death, which has recently been identified in almost all types of cancer, highlights the crucial association of iron with cancer proliferation [60,69,70,71,72,73]. In this context and in addition to ferroptosis, there are many other anticancer mechanisms involving iron metabolic pathways, proteins, and other factors, which can be targeted for anticancer activity by different anticancer drugs but also by iron-chelating drugs and metabolites such as the ACMs [18,60].

The prospect that the ACMs may be implicated in the anticancer activity of aspirin needs urgent attention and investigations. Such information, which is related to the possible effects of the ACMs on iron metabolic changes, may shed light on the possible unidentified anticancer metabolic pathways and effects of aspirin in LTLDA users. It may also shed light on related anticancer mechanisms, which could improve targeting strategies and increase the overall reduction in the incidence, as well as therapeutic outcomes in colorectal and other cancers [18,60].

## 3. The Link of Iron and Cancer and the Effects of Iron Chelators

There are many molecular, biochemical, environmental, and other factors including different forms of iron, which can trigger cancer initiation, accelerate cancer progression, and facilitate metastasis [60,74]. Furthermore, iron-associated metabolic, genomic, redoxomic, transcriptional, and other pathways, including ferroptosis, appear to play a major role in cancer cell development and could be a major contributory factor to the high overall cancer mortality rate observed worldwide [1,2,3,4,5,60,74,75,76,77,78,79,80].

The targeting of different forms of iron toxicity, associated iron factors, metabolic pathways, or cellular processes such as ferroptosis involved in cancer initiation and proliferation are considered among the latest important strategies for the design of new anticancer drugs. In this context, natural or synthetic chelators modulating iron processes involved in different aspects of cancer is the subject of many investigations and the major focus of the development of new anticancer therapeutics [60,81,82,83,84,85,86,87].

In general, there are various forms of iron, which appear to facilitate different stages and types of cancer. For example, the presence of excess iron deposition in the liver in hereditary hemochromatosis and other iron-loaded conditions has been implicated in the increased incidence of hepatocellular carcinoma [88,89,90,91]. Similarly, different chelator–iron complexes, in particular heme and nitroso-heme, which are mainly components of red meat and processed meat, respectively, have been implicated in the increased incidence of colorectal cancer [92,93,94,95,96,97,98,99]. Furthermore, the presence of iron in asbestos and other environmental toxins is another area, where iron toxicity is thought to be linked to carcinogenicity [100,101,102,103,104,105,106,107,108,109].

Many other molecular toxicity factors related to iron are considered to be linked to cancer. For example, the catalytic activity by “labile” and other forms of iron in free radical damage is widely reported as a cause of DNA damage and cancer initiation [110,111,112,113,114,115]. In such cases, the absence of sufficient and effective antioxidant mechanisms, a DNA repair system, as well as effective innate immunity may facilitate the development of cancer [116,117,118,119,120,121,122,123]. In particular, free radicals and antioxidants are widely discussed in the public domain. Similarly, many pharmaceutical and nutraceutical companies are actively engaged in the production and sale of antioxidant formulations for anticancer protection and related effects, not only through pharmacies but also many other general purpose shops and the internet, in lucrative businesses worldwide worth multi-billion euros [81].

**Figure 2:**
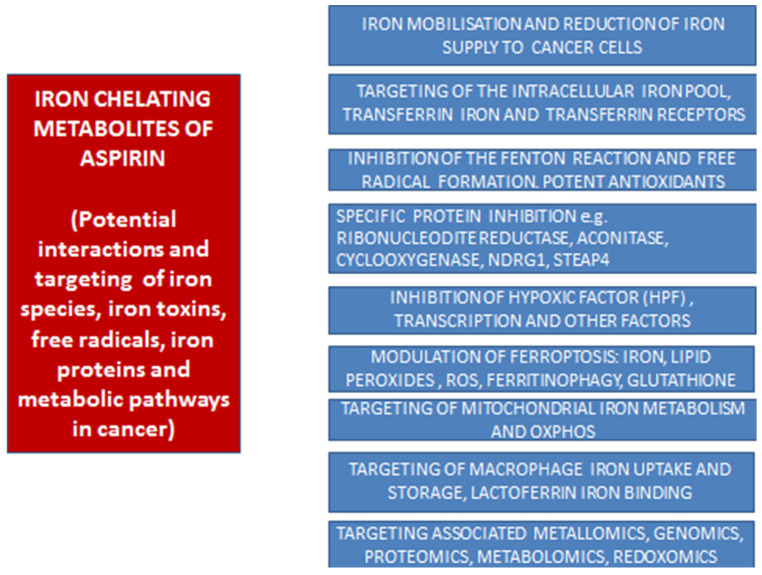
Mechanistic insights and potential iron targets for the anticancer activity of the aspirin chelating metabolites (ACMs). The targeting involves the inhibition of iron or modulation by iron chelators, such as the ACMs, of iron metabolic pathways, biological activities of biomolecules, specific organelles, and cells (including ferroptosis) related to cancer formation, proliferation, metastasis, and drug resistance, affecting almost all types of cancer. (NDRG1: N-MYC downstream-regulated gene-1; OXPHOS: oxidative phosphorylation; ROS: reactive oxygen species; STEAP4: six transmembrane epithelial antigen of prostate, family member 4).

The molecular mechanisms of free radical damage in relation to DNA and other biomolecules by iron are very complex and involve in most cases different molecules and different conditions. For example, ferrous iron and hydrogen peroxide involved in the Fenton reaction for the production of toxic hydroxyl radicals can be facilitated by reducing agents such as ascorbate or inhibited by ferric iron-chelating drugs such as deferiprone [81,124,125,126]. Furthermore, the form of iron toxicity and the target of free radical damage may differ in each case involving, for example, the deoxyribose component in DNA damage, membrane lipid damage in ferroptosis, and cardiocytes in anthracycline toxicity in cancer chemotherapy [69,124,127]. In this context, there are different iron target characteristics in each case and the efficacy of each chelator, including each ACM, depends on their molecular, pharmacological, and other characteristics, as well as their interactions with other molecules (Figure 2) [124,125,126,127].

One of the major strategies for anticancer activity in relation to iron is the inhibition of iron delivery to cancer cells, which is vital for cancer cell growth or the inhibition of iron proteins, which are also essential for different cellular-related functions [60,61,62,74,128,129]. Restriction of iron for cancer cell growth can be accomplished by mobilizing or inhibiting iron delivery by transferrin, mobilizing iron from the intracellular LMWt iron pool, from the iron storage proteins ferritin and hemosiderin, and by inhibiting the turnover of related enzymes such as ribonucleodite reductase and cyclo-oxygenase [60,61,62,129,130,131,132]. A different form of inhibition of iron proteins is through allosteric and other interactions similar to the inhibition of ribonucleodite reductase by hydroxyurea and cyclo-oxygenase by aspirin [14,15,16,74,133,134,135].

The specific inhibition of other key proteins, receptors, antigens, and factors associated with iron metabolic processes have also been targeted by chelators and other biomolecules, which are related to cancer formation, proliferation, metastasis, and drug resistance, which affect almost all types of cancer [18,60,136,137,138,139]. Such targets include, for example, transferrin and the transferrin receptors [140,141,142,143]; specific proteins such as aconitase, mitochondrial iron metabolism, and oxidative phosphorylation [144,145,146,147,148,149,150,151,152]; the metastasis suppressor N-MYC downstream-regulated gene-1 (NDRG1) [153,154]; the six trans-membrane epithelial antigen of prostate, family member 4 (STEAP4) protein [155]; the hypoxic factor (HPF), which is a transcription factor and a substrate of the iron-containing protein HIF prolyl hydroxylases (HIF PHD) [156,157,158,159,160,161,162]; several other transcription factors and key molecular factors related to iron metabolism, etc. (Figure 2) [60,163].

Iron has also been recently implicated in several other newly discovered mechanisms and processes involving all types of cancers, as well as metastasis and drug resistance. In particular, iron has been found to play an essential role in ferroptosis, which is a relatively newly discovered programmed cell death process requiring the presence of iron for the peroxidation of cell membrane lipids of the affected cells undergoing this process [69,70,71,72,73]. In this context, iron chelators such as the ACMs and iron–chelator complexes can modulate ferroptosis according to their molecular characteristics and the tumor microenvironment [60,164,165].

Similarly, iron seems to play a major role in immuno-oncology and in particular in the reduction in the ability of iron-laden macrophages to fight cancer cells [60,166,167,168,169,170,171,172]. In this context, effective iron-chelating drugs can enhance the immune function against cancer cells by reducing the iron load of macrophages and other cells of the immune system, which, for example, has been previously shown in iron-loaded patients [18,173,174,175,176,177,178,179,180,181]. Furthermore, the targeting of iron lactoferrin by chelators such as the ACMs is also an important strategy for immunomodulation in cancer, which has not yet been fully investigated [60,182,183,184].

The overall targeting of specific processes and related biomolecules associated with metallomic, genomic, proteomic, metabolomic, and redoxomic factors involved in iron function and cancer prevention or proliferation is another area where chelating drugs and chelating metabolites such as the ACMs may play an important role in the prevention and treatment of cancer [18,60]. It is anticipated that the iron chelation effects and implications on iron metabolism by aspirin and the ACMs may proceed through similar mechanisms and targeting processes for the prevention and reduction in proliferation of cancer, as previously described for other effective anticancer chelators or chelating drugs [18,60,61,87].

## 4. The Role of Iron Modulation by Aspirin Metabolites in Cancer Prevention and Proliferation

The pharmacological activity of aspirin has been recently associated with different metabolic changes in addition to prostaglandin biosynthesis and platelet aggregation, including the causes of secondary effects such as IDA and cancer chemoprevention in LTLDA users [18,37,38,39,40,41,42,43,44].

The effect of aspirin and the ACMs on iron metabolism and especially the mechanistic insights of the cause of IDA in LTLDA users, without gastrointestinal bleeding, have recently been reviewed [18]. It appears that the administration of aspirin and its metabolism in LTLDA users results in a daily available exposure to its iron-chelating metabolites, which interact with iron and iron metabolic processes, and provides antioxidant protection. This form of continuous chelation “treatment” by the ACMs is not available to other individuals or patient categories with normal iron stores receiving other drugs. However, similar continuous daily chelation treatment is only available to transfusional iron-loaded patients treated with the more efficacious iron-chelating drugs deferiprone, deferoxamine, and deferasirox [185,186,187].

Many long-term follow-up studies have suggested that there is a link between aspirin use and anticancer effects, including long-term chemoprevention, which caused a reduction in cancer incidence in LTLDA users in comparison to non-aspirin users [40,42,44,45,46,47,48,49,50,51,52,53,54,55,56]. Similarly, there are also some coincidences in relation to the number of LTLDA users who develop IDA and a reduction in cancer incidence, which amounts to about 20–30% in both cases, although no retrospective studies have been carried out to link these two secondary effects of aspirin in these two groups of affected patients.

Recently, there has been a re-assessment of epidemiological and other studies questioning the benefit of aspirin in colorectal and other cancers. However, such studies were based on many unweighted and variable parameters and other factors, which have not been included in the general statistical evaluation, such as variable aspirin dose per body weight and metabolic profile, dietary and lifestyle habits, immunological status, etc., all of which appear to influence the development of iron deficiency and cancer [18,45,188].

### 4.1. The Effects of Aspirin and Aspirin’s Chelating Metabolites on Iron Metabolic Changes and Cancer

One of the major omissions in the pharmacological activity profile of aspirin is the assessment and characterization of the biological effects of its metabolites including their high metal-chelating potential [18]. In particular, the four ACMs salicylic acid, salicyluric acid, 2,5-dihydroxybenzoic acid, and 2,3-dihydroxybenzoic acid have all been widely reported to have high efficacy in iron binding, can effect iron metabolism, and are suspected to be the cause of iron deficiency in some of the LTLDA users [18,37,38,39,61].

In iron deficiency, many endogenous and exogenous variable parameters and factors such as body weight, lifestyle, and dietary habits of LTLDA users appear to influence the effects of the ACMs in relation to the whole-body iron balance levels following the long-term use of aspirin. Similar parameters and factors also appear to influence the effect of the ACMs in relation to different types of cancer. For example, in some types of cancer such as breast and prostate cancers, the cancer cells express higher number of transferrin receptors to acquire higher quantities of iron for proliferation [60,129,140,143]. These two types of cancer and other types, which for example can be more susceptible to oxidative stress or ferroptotic cell death processes, are more likely to be affected by the iron-binding effects of the ACMs [60].

The ability of each ACM to interact with iron and iron metabolic processes depends on many physicochemical, pharmacochemical, and other parameters [18,35,36,74,81,86,187]. There are many studies reporting the formation of iron and other metal complexes by aspirin and the ACMs, as well as iron metabolic interactions, including the involvement of metabolic pathways related to anticancer activity.

In the case of aspirin, which has been shown to possess weak iron-binding properties, the formation of an iron complex was characterized and reported in one study to have pro-oxidant activity and toxicity in liver mitochondria [189,190]. However, antioxidant activity was reported implicating aspirin through the chelation of intracellular low-molecular-weight iron in an endothelial cell model, where oxidative stress damage was caused by hydrogen peroxide, and also in other models of oxidative damage [191,192,193]. Furthermore, aspirin has been implicated in the expression of the iron transport and storage proteins and associated metabolic pathways and related physiological functions [194,195]. Similarly, many other studies have also suggested that aspirin is involved in the modulation of ferroptosis, a mechanism that has been identified in almost all diseases including cancer [196,197]. In particular, the modulation of ferroptosis by aspirin has been suggested as a major mechanism for the treatment of different cancers, metastasis, and anticancer drug resistance [197,198,199,200,201,202,203,204].

The impact of the ACMs in the anticancer activity of aspirin treatment is crucial, considering that aspirin is rapidly and almost completely metabolized to ACMs in vivo and considering that the anticancer effects are observed following many years of exposure to the drug and mostly to its metabolites. In this context, more information on iron binding and related iron removal, antioxidant and other effects in relation to anticancer activity should be examined for each ACM: salicylic acid, 2,3-dihydroxybenzoic acid, 2,5-dihydroxybenzoic acid, and salicyluric acid.

In the meantime, many non-clinical studies and clinical trials have already been carried out with most of the ACMs due to their natural occurrence as plant products (phytochelators) and their presence in many vegetarian foods, where some potential therapeutic effects have been previously identified [18,86]. In some studies, the pharmacological monitoring and concentration estimation of ACMs was carried out from the ingestion of vegetarian meals and compared to the low-dose aspirin (75–100 mg). Overall, it has been shown that there are much lower levels of the ACMs in vegetarian meals in comparison to those observed in LTLDA users [32,205,206,207,208]. However, it should be noted that many other phytochelators similar to the ACMs are present in vegetarian diets with strong iron-binding and antioxidant activity and anticancer potential [86].

Many therapeutic and other clinical effects including anticancer activity are reported for each ACM. The mechanism of the anticancer effects of salicylic acid were of particular interest, considering the rapid metabolism of aspirin following its oral administration and transformation into this initial main metabolite, which in many cases makes it difficult to separate the biological and clinical activities between the two [19,20,209,210]. Such questions became crucial, especially since many clinical findings have suggested that LTLDA can prevent colorectal cancer, other cancers, and metastasis. In particular, the findings from such long-term monitoring studies have raised questions as to whether iron chelation and other properties of salicylic acid are implicated in the mode of its anticancer activity in colorectal cancer and other cancers [33,34,56,210,211,212,213,214,215,216,217,218,219,220,221,222].

Another aspirin metabolite and naturally occurring plant and mammalian product with iron-chelating properties, which is widely reported to have anticancer, anti-inflammatory, antioxidant, antimicrobial, hepatoprotective, neuroprotective, and other similar beneficial health effects, is 2,5-dihydroxybenzoic acid or genticic acid [223]. In particular, 2,5-dihydroxybenzoic acid has been suggested to act as a mammalian siderophore (iron chelator) in innate immunity against infection by competing with microbial siderophores [224,225,226,227,228,229,230]. Similarly, many studies using various models have suggested that 2, 5-dihydroxybenzoic acid can also exert anticancer effects via different mechanisms and under different experimental conditions [231,232,233,234,235,236].

The naturally occurring compound, phytochelator and siderophore 2,3-dihydroxybenzoic acid or 2-pyrocatechuic acid, is the most widely known and clinically studied of the four ACMs, especially for its iron-chelating properties in the treatment of iron overload in thalassemia [18,237,238,239,240]. Hundreds of in vitro and in vivo preclinical investigations have been reported for this aspirin metabolite, including structure/activity correlations, in vitro and in vivo toxicology and clinical trials regarding its iron-binding properties and increasing iron excretion effects [241,242,243,244,245,246,247,248,249,250,251]. Despite that 2,3-dihydroxybenzoic acid amounts to about 1–2% of the ACMs, many antioxidant effects have been reported, including its increase in production/biotransformation from aspirin during increases in oxidative stress conditions and increased hydroxyl radical production [252,253]. Similarly, many investigations regarding the anticancer properties of 2,3-dihydroxybenzoic acid, have confirmed the anticancer potential of this ACM and its homologue 3,4-dihydroxybenzoic acid or protocatechuic acid in different experimental models [87,236,254,255,256,257,258,259,260,261,262,263,264,265].

Further studies are needed to examine the anticancer effects of the ACMs including salicyluric acid (or salicylglycine or 2-hydroxyhippuric acid), which is quantitatively the highest in concentration of the excreted ACMs, and for which few reports are available so far in relation to iron or other metal chelation and potential anticancer activity [266,267,268,269,270,271,272,273,274,275,276].

Overall, aspirin and ACMs appear to play a significant role in iron metabolism and associated physiological processes including several pathways involved in iron chelation and related anticancer activity. In particular, the ability of 2,3-dihydroxybenzoic acid and the other of ACMs to mobilize iron from cells and from iron-loaded tissues of thalassemia patients suggest that the ACM have high potential for specific targeting of biomolecules related to cancer formation, drug-resistance, metastasis, and migration within cancer tissues [241,242,243,244,245,246,247].

### 4.2. The Antioxidant Effects of the Aspirin Iron-Chelating Metabolites

There is a broad spectrum and wide diversity in the molecular and redox properties of metal ions, chelating drugs, and chelator–metal complexes. Similarly, there is increasing prospect for the development of a wide range of therapeutic targeting and strategies, as well as for use as antioxidants or redox modulators in many diseases of free radical pathology, including cancer [74,81,83,84,85,86]. In this context, anticancer targeting based on redox activity including the antioxidant and pro-oxidant interactions of metal ions, chelators, and chelator metal complexes with different types of biomolecules is a rapidly growing and developing area in cancer therapeutics [74,81,129,277,278,279,280].

In biological systems, redox activity is mostly based on iron and copper catalysis, both of which have variable properties and effects under different conditions in health and disease and functional differences related to various types of cells including normal and cancer cells [60,74]. Redox homeostasis is maintained under normal conditions due to the presence of antioxidant systems involving antioxidant enzymes, antioxidant endogenous and exogenous (dietary) molecules, and the presence of repair systems. In this context, targeting proteins, metabolic pathways and processes involving iron or copper and of other related proteins, metabolic pathways, and processes involved in the utilization and metabolism of free radicals, are important parameters in anticancer therapeutic strategies involving redox activity [68,74,81].

Metal chelation can have variable effects on redox homeostasis. Some of the mechanistic insights of the anticancer activity of chelators and their metal complexes can be illustrated from the variable effects of three major classes of chelating drugs, namely anthraquinones, e.g., doxorubicin, where iron binding is implicated in its toxicity of oxidative stress damage; thiosemicarbazones, e.g., triapine, where iron potentiate its anticancer effects; and alpha-ketohydroxypyridines e.g., deferiprone, where iron binding prevents oxidative damage [81,124,251,281,282,283,284,285]. Variable interactions and effects of the above three classes of drugs on redox homeostasis is also observed in the presence of naturally occurring plant products (phytochelators) such as vitamin C or ascorbate. Such interactions and effects are continuous due to the presence of vitamin C in different food products, which are consumed daily, resulting in pro-oxidant or antioxidant activity under different conditions [124,125,126]. In particular, vitamin C is widely advertised in the nutraceutical industry as antioxidant plant nutrient with many therapeutic effects including the prevention and treatment of cancer, where it was investigated in many preclinical studies and many clinical trials [286,287,288,289,290].

**Figure 3:**
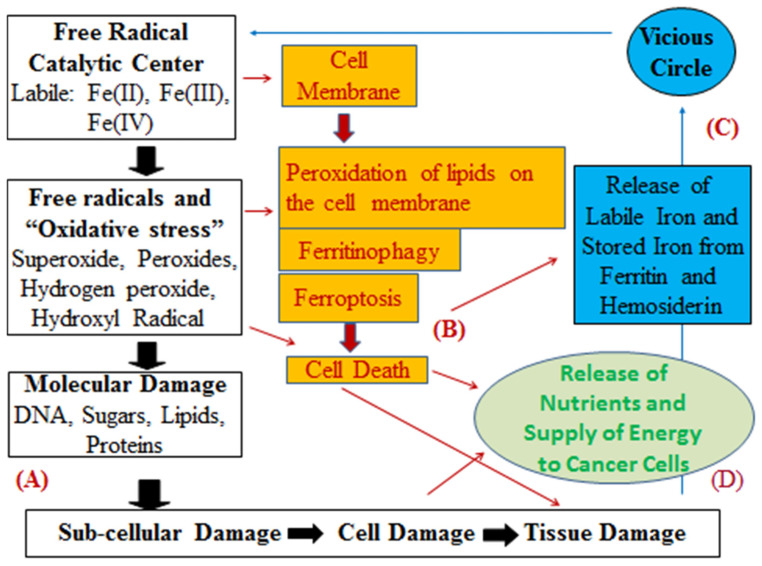
Mechanistic insights into the catalytic role of iron in free radical pathology and ferroptosis. Iron catalyzes the formation of free radicals, causing oxidative stress leading to molecular, subcellular, cellular and tissue damage (A). A similar pathway is followed in ferroptosis where iron catalyzes the formation of free radicals causing lipid peroxidation on the cell membrane and cell death (B). Cell and tissue damage and ferroptotic cell death cause the release of more intracellular iron resulting in a vicious circle of free radical production (C). Similarly, cell and tissue damage cause the release of nutrients and other biomolecules, which can be utilized by cancer cells for energy production and for proliferation (D).

The targeting of oxidative stress toxicity damage, which is mostly catalyzed by iron has been identified in almost all diseases including cancer (Figure 3) [68,81,279,291,292]. In general, chelators which can have access and bind strongly iron at the iron catalytic center can potentially inhibit this toxicity [71,84,86,292]. In this context, the potential inhibition by the ACM of the iron catalytic centers causing this form of toxicity could provide a sustainable and continuous method of the therapeutic activity of aspirin and ACMs in LTLDA users, and overall lead to chemoprevention and inhibition of cancer proliferation (Figure 3).

There are many reports on the antioxidant activity of the ACMs. For example, 2,3-dihydroxybenzoic acid has been studied in different models of oxidative stress toxicity, where iron chelation was considered as the major mechanism of the prevention of oxidative damage (Figure 3) [18,127,250,251,291,292]. These studies have suggested that the level of the antioxidant activity by 2,3-dihydroxybenzoic acid was concentration dependent. The antioxidant activity of 2,3-dihydroxybenzoic acid has also been reported in different other models of oxidative stress and under different experimental conditions [18,236,237,252,253].

In the meantime, an intriguing major antioxidant pathway based on iron chelation appears to be initiated following the oral administration of aspirin and its metabolism to the ACMs. In particular, increased production of 2,3-dihydroxybenzoic acid and 2,5-dihydroxybenzoic acid has been reported as a marker of aspirin metabolism in inflammatory and other conditions, which is associated with increased production of hydroxyl radical formation during oxidative stress [252,253,293,294]. In many such cases the increased production of these two ACMs was implicated, presumably through iron chelation, in the increased antioxidant effects observed during the administration of aspirin in inflammatory and other similar conditions [252]. In this context, many clinical and in vivo studies examining the antioxidant effects of 2,3-dihydroxybenzoic acid and 2,5-dihydroxybenzoic acid in several diseases have been carried out using aspirin as a pro-drug [18]. Overall, 2,3-dihydroxybenzoic acid and 2,5-dihydroxybenzoic acid appear to be a generally safe and effective iron chelators for increasing iron mobilization and for promoting antioxidant activity [18].

The relationship of aspirin’s anti-inflammatory activity and its antioxidant potential or that of the ACMs has not yet been fully clarified, despite that some studies have previously suggested there may be a link between these two processes [189,191,192,199,295,296,297]. In addition, the redox effects of all of the ACMs have not yet been thoroughly investigated in relation to their iron-binding potential and the Fenton reaction, where iron is the catalytic center for the formation of toxic hydroxyl radicals. It is worth noting that previous studies have shown that deferoxamine, deferiprone and similar iron chelators inhibited free radical reactions and the activity of the iron-containing proteins cyclo-oxygenase and lipoxygenase [18,131,132]. The mode of chelating activity in the latter case was suggested to be the mobilization of iron by the chelator and reduction of available iron for the turnover of cyclo-oxygenase and lipoxygenase activity [71,131,132,292]. In this context, further studies are needed to investigate the partial inhibition of cyclo-oxygenase and lipoxygenase through iron chelation in relation to the overall anti-inflammatory activity of aspirin and the ACMs, as well as their pharmacological implications in other diseases.

Another major unexplored area of great importance in pharmacology and medicine are the effects of aspirin and ACMs, as well as their iron complexes on ferroptosis (Figure 3) [69,70,71,72,73]. In this context, several preliminary investigations have already implicated aspirin in the process of ferroptosis via different mechanisms [191,195,196,198,202,204]. In addition, the interaction of aspirin, ACMs and their iron complexes in ferroptotic cell death may also have wider implications in the therapeutic outcome of aspirin in cancer and many other related diseases associated with ferroptosis.

Further investigations are needed to identify the precise role(s) of aspirin and the ACMs in ferroptosis and associated diseases, considering that ferroptosis is regulated by iron and reactive oxygen species, both of which can be modulated by the iron chelation and antioxidant properties of the ACMs.

### 4.3. New Mechanistic Insights into the Role of Aspirin and Its Metabolites in Colorectal Cancer

Colorectal cancer is one of the rapidly expanding forms of cancer, especially at the younger ages (50 years>). It is the third most common cancer and second in mortality, responsible for about one million deaths per year, with poor prognosis and without available effective treatment in many cases, despite the colossal efforts for its prevention and of new therapies [298,299,300,301,302,303,304]. In this context and based on a risk/benefit assessment, any nutraceuticals, new experimental drugs and repurposed drugs are granted accelerated approval for clinical investigations for use in colorectal cancer and similar diseases [5,305,306].

In the last few years, the rise in the numbers and mortality rate of colorectal cancer patients raised the alarm for instant action by many national health authorities. In particular, the increased survival and reduced risk in colorectal cancer observed in 20–30% of LTLDA users, prompted the USA drug regulatory health authorities to recommend in 2016 the use of LTLDA for the chemoprevention of colorectal cancer in people of 50–59 years of age [40,41,42,43,44,45,46,47,48]. However, a new report by the same USA authorities in 2022 has suggested that new epidemiological results indicated that the effects of aspirin on colorectal cancer were “less robust and highly variable” [45,307,308]. This latter decision has been questioned by many investigators because of the lack of sufficient pharmacological and epidemiological parameters required for the comparison of the use or no use of LTLDA in colorectal cancer. In particular, more recent (2024) findings, following the detailed national epidemiological studies in Norway and the USA and by other investigators elsewhere strongly recommended again the use of LTLDA for the prevention of colorectal cancer [309,310,311]. The recent 10 year monitoring study in the USA considered many unweighted factors in LTLDA users and the authors concluded that aspirin was especially beneficial for LTLDA users with unhealthy lifestyle including body mass index parameters, unhealthy diet, alcohol intake, low physical activity, and smoking [310]. Similar studies in the UK and elsewhere suggested that the use of LTLDA is likely to benefit particularly individuals with family history of different cancers and other patient sub-groups [311,312,313,314].

The cause of colorectal cancer is also largely unknown, despite that most epidemiological studies have linked colorectal cancer mostly to dietary habits, in which case the rate of incidence is much lower in vegetarian populations in comparison to meat eating populations [315,316,317,318,319]. In particular, many epidemiological studies and clinical trials have suggested that dietary habits with regular use of red meat food products and especially processed meat food products containing the iron complexes heme and nitroso-heme respectively, as well as other possible toxic iron complexes in food, are mainly responsible for the carcinogenic effects leading to the development of colorectal cancer [92,93,94,95,96,97,98,99].

Different molecular mechanisms of toxicity related to heme, nitroso-heme, and other iron complexes have been suggested on the molecular level including oxidative stress toxicity, leading to DNA damage and changes, which can progressively lead into cancer initiation and proliferation [77,320,321,322,323,324]. Similar molecular mechanisms may be initiated from the metabolic pathways involved in ferroptotic cell death in the GIT, where iron and free radicals play the major roles [69,70,71,72,73,74,325,326,327]. Additional contributory toxicity factors include the lipophilicity of heme, nitroso-heme, and other similar toxic iron complexes leading to the accumulation in affected cells and in long-term persistent toxicity, facilitating carcinogenicity [74,86,328,329].

Many personalized and other factors may contribute to the level of toxicity exerted by heme, nitroso-heme, and other similar toxic iron complexes in the GIT. Such factors include the rate of iron absorption and excretion from the GIT in each individual. In this context and under normal physiological conditions, the shedding of enterocytes in the gut lumen every few days may contain additional non-absorbed heme, nitroso-heme, or other toxic iron species contributing to the overall iron toxicity and carcinogenicity [60,74,129,291,328,329]. Similarly, several other contributory toxicity mechanisms may also be involved related to the low absorption of iron from the GIT, which for example may be caused by diseases or through the inhibition of ferroportin by the excess production of hepcidin leading eventually to the lower availability of iron to the tissues and to anemia [74,328,329]. Furthermore, many drugs including iron supplements, dietary and other factors may increase excess iron in the GIT and exacerbate iron toxicity thus promoting carcinogenicity including the development of colorectal cancer [74,328,329].

The presence of excess or toxic forms of iron is a negative prognostic factor not only for colorectal cancer but also for all other diseases [330]. In this context, the time period of exposure to the iron toxins residing in the GIT is a major factor leading to carcinogenesis [74,329,330]. This parameter is of great significance in the reduction of incidence of colorectal cancer, considering that health authorities and nutrition professionals promote the use of vegetarian food, which for example is high in fiber and antioxidants, for facilitating the rate of defecation and reducing constipation, allowing the clearance of excess toxic components including iron and free radicals in feces [331,332,333].

The role of microbiota is also very important in relation to the initiation and proliferation of colorectal cancer and other diseases of the GIT, as well as iron metabolism and iron toxicity in the GIT [328,334,335,336,337,338]. In particular, interactions of microbiota with food components and drugs may potentiate or reduce their activity and toxicity. For example, selected bacterial constituents of microbiota may metabolize heme and nitroso-heme by heme oxygenase converting them to non-heme iron, carbon monoxide, and bilirubin with implications on the overall toxicity and carcinogenicity affecting the GIT [339,340,341].

The chemoprevention and anticancer effects of aspirin in colorectal cancer suggests that there is a major possibility that the drug and its metabolites express their pharmacological and anticancer activity both in the GIT and systemically. In this context and considering that only about 60–70% of aspirin is absorbed following oral administration, most of the remaining non-absorbed aspirin may be pharmacologically active. Furthermore aspirin may also be metabolized to the ACMs by the host and bacteria in the GIT, affecting among other iron homeostasis and anticancer activity in the GIT [18,33,255,342,343]. Microbiota and other endogenous or exogenous factors may also influence the metabolism and pharmacological activity of aspirin in the GIT and its conversion to the ACMs [328,331,332,333,334,335,336,337,339]. In this context, iron chelation by ACMs may also affect iron metabolism, absorbable iron levels and the overall anticancer activity in GIT.

It appears from many pre-clinical and clinical findings, as well as epidemiological studies that the administration of aspirin results in a threshold of active anticancer ingredients in the GIT including sufficient concentration of ACMs, which are required for achieving the reduction or modulation of iron toxicity in carcinogenicity and cancer proliferation.

Overall, it can be proposed that the anticancer targeting mechanism by aspirin and the ACMs may involve the reduction or modulation of toxic iron species and complexes such as heme and nitroso-heme toxicity, which are considered the major causes of colorectal cancer. In particular, the ACMs may interact with heme and nitroso-heme and microbiota heme oxygenase for the conversion of heme and nitroso-heme to non-heme iron forms that can be chelated by ACMs and render iron non-toxic for promoting colorectal cancer. The anticancer effects of aspirin and ACMs in colorectal cancer could also be exerted systemically through the blood circulation in addition to the microtumor environment in the GIT. This mode of action resembles a pathway similar to the anticancer chemotherapy options offered for colorectal cancer patients systemically by other drugs.

The mechanisms of iron chelation through the ACMs, which are involved in the prevention of oxidative stress and other forms of iron toxicity, as well as pathways such as ferroptosis may also be implicated in patient prognostic improvements observed not only in colorectal cancer but also in other cancers and other disease categories of LTLDA users (Figure 3) [18,44,47,60,61,74,81].

Further studies are needed to confirm in addition to the anticancer activity, the anti-metastatic effects observed by aspirin and the ACMs, as well as their effects on drug resistance, which are the major causes of mortality in cancer patients [18,53,54,234,261,262,265,344]. In particular, the interactions of aspirin and the ACMs with ferroptosis and associated effects on metastasis and drug resistance may increase their anticancer therapeutic potential and prospects [345,346,347,348].

## 5. Future Investigations and Limitations in the Use of Aspirin in Cancer

**Figure 4:**
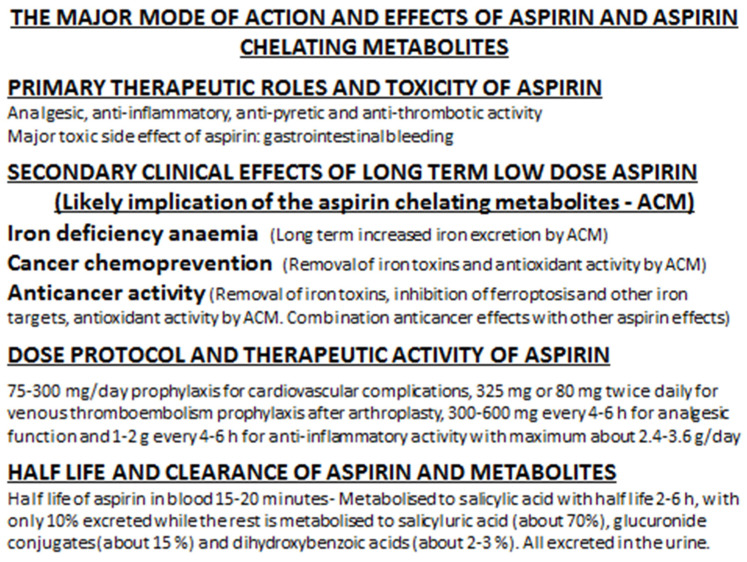
The major pharmacological characteristics and clinical effects of aspirin and its chelating metabolites, including the anticancer activity observed in long-term, low-dose aspirin (LTLDA) users. The secondary clinical effects of iron deficiency anemia (IDA), cancer chemoprevention and anticancer activity in LTLDA users appear to be related primarily to the pharmacological characteristics of the aspirin’s chelating metabolites (ACMs).

Hundreds of millions of people worldwide are taking aspirin daily for many therapeutic purposes (Figure 4). Among them are many millions of the elderly population who have been using low-dose aspirin daily for many years as prophylaxis, mainly against cardiovascular disease. Epidemiological evidence has suggested that about 20–30% of LTLDA users after many years of daily use of aspirin appear to develop IDA and about the same number to have a lower risk of developing colorectal cancer and possibly other cancers. In the meantime, there are thousands of publications referring to experimental and clinical evidence related to the use of aspirin as a chemopreventative and anticancer agent. Similarly, there are many proposals suggesting the involvement of different anticancer mechanisms, metabolic pathways, and molecular effects, not only by aspirin itself but also by its metabolites (Figure 4) [6,7,8,9,10,11,12,13,14,15,16,17,18,37,38,39,40,41,42,43,44,45,254,255].

Despite that there is a general consensus regarding the analgesic, anti-inflammatory, anti-pyretic, and anti-thrombotic activity of aspirin, none of the proposals regarding the mechanisms and causes of the secondary clinical effects of aspirin in LTLDA users, such as IDA and the prevention of colorectal cancer, are generally accepted [8,9,10,11,12,13,14,15,16,18]. Furthermore, the risk/benefit assessment of the use of aspirin for the prevention of colorectal and other cancers in comparison to the risks of aspirin-associated gastric toxicity and other secondary effects such as IDA are also still considered under investigation (Figure 4) [349,350,351]. In this context, it is anticipated that patients with major risk of developing a high level of toxicity from the side effects of aspirin use, such as serious gastric bleeding and high levels of sideropenia, could be excluded from the prophylactic use of LTLDA in colorectal cancer.

There are many other limitations that could affect the anticancer effects and strategies of aspirin in different categories of LTLDA users. Some of these limitations and corresponding effects are related, for example, to differences in the dose and formulation of aspirin use, dietary habits, physical activity, the effects of the underlying condition, ADMET, and other characteristics of aspirin users, etc. [18,310,311,351]. Such limitations may be critical in achieving optimal anticancer activity during LTLDA use. For example, the widely proposed dose of about 75 mg in LTLDA users is arbitrary in most cases, since it is intended for people with a body weight of 70 kg. However, for people with a body weight lower than 70 kg, the dose is higher in standard mg/kg posology, and for obese or people with greater than 70 kg of body weight, the above dose of aspirin is low and may not achieve the desired therapeutic aspirin threshold or the corresponding ACM concentration required for anticancer activity [18,352,353]. Differences in the rate of metabolism and the biotransformation of aspirin to variable levels of each ACM in LTLDA users may also affect the overall anticancer and other therapeutic effects of aspirin [18].

The identification of different sections or categories of patients to be mostly benefited from the use of LTLDA, as well as contributory factors that may enhance the anticancer activity of aspirin, are not currently fully understood [310,311,312,313,314]. For example, vegetarian patients may be at an advantage over other categories of patients because of the presence of higher levels of ACMs in blood and tissues in comparison to non-vegetarians, due to the natural occurrence of ACMs in fruit and vegetables in vegetarian diets [18,86,205,206,207,208]. In contrast, vegetarian patients using LTLDA have a higher risk for developing IDA than non-vegetarians (Figure 4) [18].

The latent period for confirming the anticancer activity of aspirin may be another crucial parameter, which has not yet been clearly defined. For example, in some cases, the therapeutic benefits of low-dose aspirin against cancer and other diseases may be confirmed after 5 years or more of daily use, but not earlier [310,349]. It should be noted that similar effects and implications over this time period have been observed from the posology and time period of LTLDA use in patients who developed IDA. In such cases, the overall iron-mobilizing effects of the ACMs are like in any other chelator cases, which are dependent on the concentration and rate of iron mobilization [18,352]. Similarly, it appears that aspirin and its chelating metabolites provide in LTLDA users long-lasting anticancer effects from its daily or near-daily regular use.

The important question as to whether there is a connection between IDA and anticancer activity is also still unanswered. Although the occurrence of IDA and anticancer effects have a similar latent period and about the same proportion of LTLDA users are affected, there are no studies or reports suggesting that there is a clear link between these two aspirin secondary clinical effects or that the same individuals from these groups are affected by both of these secondary aspirin effects. However, it is highly unlikely that some of the mechanisms of activity are not common to both of these secondary aspirin effects and that some such mechanisms may involve iron chelation and modulation of the iron effects resulting in anticancer activity (Figure 4) [18,60].

Many other factors and parameters may also modulate aspirin and ACMs’ anticancer potential. More studies are needed, for example, on the effects of aspirin and ACMs on other essential metal ions including zinc, copper, and calcium, which may have important implications for the general mode of anticancer activity and other effects of aspirin [57,63]. On the clinical level, co-administration of iron formulations and other metal supplements with aspirin should be avoided because of interactions that may result in the negation of their therapeutic effects including aspirin’s and ACMs’ anticancer activity.

Further investigations are also needed regarding the toxicity of iron arising from heme and nitroso-heme, which are the primary suspected toxins implicated in the cause and progression of colorectal cancer. In such cases, the anticipated intervention and modulation of the toxicity by ACMs and other chelators may result in the reduction of the carcinogenic effects of these heme molecules. However, different mechanisms of interactions have been previously shown by the iron-chelating drugs deferiprone and deferoxamine, maltol, hydroxyurea, and other chelators involving different forms of iron and metabolic pathways [18,60,61,86,124,125,126,127,128,129]. In particular, investigations into the role of the induction of ferroptosis and carcinogenicity by heme and nitroso-heme may shed more light on the cause of colorectal and other cancers. In contrast, the inhibitory role of the ACMs in such processes, similar to other chelators, may prove critical for identifying new aspirin-related anticancer mechanisms [60]. Similarly, the effects of aspirin and ACMs in different types of cancer, cancer stem cells, metastasis, and drug resistance could also shed more light on the mechanisms of the overall anticancer activity of aspirin.

Future aspirin therapies against cancer could be developed based primarily on the pharmacological, iron-chelating, and antioxidant properties of the ACMs. In such cases, the development of novel aspirin-based treatment strategies against different types and stages of cancer should consider the role of aspirin as a pro-drug, which generates a combination therapy through several iron chelator/antioxidant/anticancer metabolite molecules. The mobilization of iron by the ACMs could inhibit cancer cell proliferation, metastasis, and immune escape [60]. Aspirin and the ACMs could also be used as an adjuvant or synergistic anticancer therapies in combination with other chelating or anticancer drugs, especially in relation to the drug resistance of established therapies and for refractory and recurring tumors in almost all types of cancer, metastasis, and drug resistance [60,197,198,199,200,201,202,203,204].

Several other future therapies could be developed based on iron metabolic processes affected by the ACMs, including the modulation of the activity of the iron protein aconitase, which plays a major role in mitochondrial metabolism and function, which is crucial for tumor proliferation, metastatic tumor cells, chemotherapy, radiotherapy-resistant tumor cells, and cancer stem cells [60,144,145,146,147,148,149,150,151,152,197,198,199,200,201,202,203,204]. Similarly, ACMs targeting the modulation of NDRG1 involved in the regulation of metastasis can play a major role in inhibiting cancer progression and metastasis [60,153,154,197,198,199,200,201,202,203,204] (Figure 2). Furthermore, aspirin through iron chelation by ACMs could play a crucial therapeutic role in all types and stages of cancer, especially in cases where ferroptosis is identified to be the major form of cell death [201].

Future improved aspirin chemoprevention therapies based on effective aspirin posology could also be developed using effective LTLDA protocols. In such cases, improved continuous antioxidant therapy for prophylaxis against cancer could be provided in LTLDA users, based on the increased antioxidant capacity of the ACMs and in combinations with dietary changes and other antioxidants or drugs. In particular, initial clinical trial results suggest that aspirin may play an active role in enhancing immunosurveillance in the tumor microenvironment against colorectal cancer [354].

In the meantime, new discoveries and new developments in all areas related to cancer have increased the prospects for better understanding anticancer mechanisms and therapeutic activity. For example, new diagnostic methods for monitoring colorectal, prostate, and other cancer progression could play significant role in the assessment of the long-term treatment effects of aspirin and other drugs with potential anticancer activity. Similarly, epidemiological, pharmacological, and other studies are also needed within the concept of personalized medicine. This concept may include individual lifestyle and other characteristics such as immunological status, DNA repair capacity mechanisms and tumor suppressor p53 levels, the effect of combination therapies of aspirin and ACMs with other dietary, environmental, and microbiota molecules, and various other drugs in different categories of patients [340].

## 6. Conclusions

The omission of the possible role of the aspirin metabolites and especially the ACMs in anticancer activity and in the cause of IDA is a major setback in the overall pharmacological assessment of the role of aspirin in these conditions. In particular, the rapid metabolic transformation of aspirin and the quantitative production of metabolites with much longer half-lives than aspirin, suggests that the ACMs may play a predominant role in these secondary effects of aspirin use. The iron-chelating potential of each ACM, but also their combination, which resembles iron chelation combination therapy, is likely to be involved in anticancer activity through the targeting of iron involved in free radical damage, in iron toxins involved in carcinogenicity, in iron proteins and associated metabolic pathways including ferroptosis associated with cancer, and other similar mechanisms. Most importantly, the ACMs appear to be involved in the mitigation of the toxicity of heme, nitroso-heme, and possibly other iron species, which are considered to be the major cause of colorectal cancer. Overall, the mode of action of aspirin as a pro-drug of several metabolites with iron-chelating, antioxidant, and anticancer potential, present in the body almost on a daily basis, appears to offer LTLDA users prophylaxis for colorectal and other cancers, and also other diseases. 

It is suggested that the therapeutic effects of aspirin in cancer appear to depend primarily on the activity of the ACMs. Other anticancer mechanisms may also play a role and may act synergistically with the ACMs. The overall assessment of the pharmacological role of aspirin in cancer and other diseases is incomplete without taking into consideration its rapid biotransformation, and the much longer half-life activity of its metabolites, especially the ACMs in blood and other tissues. Similarly, epidemiological, pharmacological, and other assessments of aspirin are incomplete without taking into consideration several parameters for its pharmacological evaluation in LTLDA users such as dietary habits, ADMET characteristics, and the effects of the ACMs.

Further investigations are needed for the determination of aspirin’s posology, the therapeutic index of each ACM, and the latent period of LTLDA use for preventing cancer proliferation. Epidemiological and pharmacological studies are also needed for the identification of individuals who are most likely to benefit from LTLDA use and from combination therapies of aspirin and ACMs with other drugs, dietary changes, and microbiota molecules. The mode of action of aspirin as a chelating antioxidant pro-drug appears to be unique in medicine and requires further investigation.

## Abbreviations

ACM	aspirin chelating metabolite
ADMET	absorption: distribution, metabolism, elimination, and toxicity
GIT	gastrointestinal tract
HIF	hypoxia-inducible factor
HIF PHD	hypoxia-inducible factor prolyl hydroxylases
IDA	iron deficiency anemia
LTLDA	long-term, low-dose daily aspirin
MRI	magnetic resonance imaging
NDRG1	N-MYC downstream-regulated gene-1
OXPHOS	oxidative phosphorylation
ROS	reactive oxygen species
STEAP4	six transmembrane epithelial antigen of prostate, family member 4
